# Combined patterns of physical activity, screen-based sedentary behaviour, and sleep duration and their associations with depressive symptoms in Chinese adolescents

**DOI:** 10.3389/fpubh.2025.1691683

**Published:** 2025-11-18

**Authors:** Yi Lin, Jin-Ying Huang, Richard Rankin, Stuart McDonald, Dan-Jie Jiang, Si-Xuan Li, Wang-Wei Lou, Yi Chen, Qing-Hai Gong

**Affiliations:** 1 Faculty of Humanities and Social Sciences, University of Nottingham Ningbo China, Ningbo, Zhejiang, China; 2 College of International Economics & Trade, Ningbo University of Finance & Economics, Ningbo, Zhejiang, China; 3 Faculty of Science and Engineering, University of Nottingham Ningbo China, Ningbo, Zhejiang, China; 4 Ningbo Municipal Center for Disease Control and Prevention, Ningbo, Zhejiang, China; 5 Ninghai County Center for Disease Control and Prevention, Ningbo, Zhejiang, China

**Keywords:** physical activity, screen-based sedentary time, sleep duration, healthy lifestyle, sedentary behaviour, depressive symptoms, adolescents

## Abstract

**Background:**

Depression is a growing public health burden and is one of the leading causes of disability and illness in adolescents. Lifestyle factors are associated with depressive symptoms (DSs) in adolescents. This study aimed to estimate the prevalence of DSs and investigate the independent and combined associations between DSs and moderate-to-vigorous physical activity (MVPA), screen-based sedentary time (ST), and sleep duration (SLD) among Chinese adolescents aged 11–19 years.

**Methods:**

Repeated cross-sectional data were collected through questionnaires on health status and influencing factors for high school students in Ningbo, China, from a school-based study conducted from September to October in 2022 and in 2023. A multistage, stratified cluster sampling procedure was used to select target adolescents aged 11–19 years. DSs were assessed using the Centre for Epidemiological Studies Depression Scale. A multivariate analysis was used to assess the associations between independent lifestyle behaviours (MVPA, ST, and SLD) and the combined patterns of those lifestyle behaviours with DSs.

**Results:**

The prevalence of DSs amongst the adolescents was 16.3%. Low MVPA, excessive ST, and short SLD were independently associated with a greater likelihood of DSs (all *p* < 0.001). The unhealthiest combined pattern of low MVPA, excessive ST, and short SLD was associated with the highest odds of DSs (OR:4.2, 95% CI:3.3, 5.2, *p* < 0.001). In addition, the other combined patterns of MVPA, sleep duration, and ST were significantly associated with an increase in DSs compared with the healthy combined patterns of high MVPA, appropriate ST, and sufficient SLD.

**Conclusion:**

Our results indicate that MVPA, ST, and SLD were independently and in combination associated with an increase in DSs. Effective school-based health education programmes aimed at promoting healthy lifestyles are necessary to protect the mental health of Chinese adolescents. Future longitudinal studies are needed to confirm causality between the combined lifestyle patterns and DSs in Chinese adolescents.

## 1 Introduction

Depression, as a depressive disorder, is one of the most challenging public health concerns in the world. It is considered a serious mental disorder that manifests as symptoms of low mood, loss of pleasure, and aversion to activity, resulting in low quality of life, disability, and a higher likelihood of premature death (1, 2). Adolescence, defined as a period ranging between 10 and 19 years of age, is a transitional stage of changes in hormone levels and body composition, and rapid physical, cognitive, and psychosocial development during the period of life between childhood and adulthood (3). The global prevalence of depression and elevated depressive symptoms (DSs) amongst adolescents increased from 24% between 2001 and 2010 to 37% between 2011 and 2020 (4), and this rate increased with age (5). A recent meta-analysis of observational studies involving 29,626 Chinese children and adolescents reported that approximately 19.9% of students had DSs, with estimates of 17.8%, 23.7%, 22.7%, and 14.5% in the eastern, central, western, and northeastern regions of China, respectively (6). The long-term effects of depression and DSs can persist far beyond adolescence and are associated with a higher risk of psychological and mental health problems in adulthood, such as self-harm and suicide (7, 8).

Adolescence is an important period for the development of healthy lifestyle behaviours associated with mental health (9). However, unhealthy lifestyle behaviours, including low levels of physical activity (PA), excessive sedentary behaviour (SB), and insufficient sleep duration (SLD), are considered ‘invisible’ risk behaviours that can cause psychological problems in children and adolescents (10, 11). Given the rapid development of economies and technology over the past five decades in China, the use of electronic devices has increased dramatically nationwide. Consequently, Chinese adolescents have followed global trends in low-quality lifestyles. According to previous Chinese studies, 77.3% of students aged 9–18 years engaged in less than 60 min of moderate-to-vigorous physical activity (MVPA), 85.8% of students aged 6–17 years engaged in screen-based sedentary time (ST) longer than 2 h/day, and 57.2% of students aged 8–16 years had insufficient SLD (12–14).

There is growing interest in the association between health and lifestyle behaviours. Evidence shows that lifestyle factors, including a higher frequency of PA, short ST, and sufficient SLD, can independently benefit psychological health in children and adolescents (10, 15, 16). A systematic review examining the associations between combinations of PA, SB, and SLD and mental health problems across 12 countries indicated favourable associations between meeting all three recommendations and better mental health indicators amongst children and adolescents (17). These combined lifestyle behaviours play an important role in the prevention of mental disorders. Therefore, it is necessary to conduct further studies to better understand the interactions between PA, SB, and SLD and their associations with DSs in adolescents.

An increasing number of studies have linked lifestyle behaviours to mental health. Most studies investigated PA, ST, and SLD separately (18–20). In addition, studies on the combination of PA, ST, and SLD and their relationship with DSs are still lacking in China, and the combined associations of these lifestyle behaviours with DSs remain unclear. Therefore, this study aimed to (1) estimate the prevalence of DSs in Chinese adolescents aged 11–19 years in Ningbo, Zhejiang Province, (2) examine the independent relationship between MVPA, ST, and SLD and DSs, and (3) investigate how combined lifestyle patterns associate with DSs.

## 2 Methods

### 2.1 Study design and study population

Two repeated school-based cross-sectional studies in Ningbo, Zhejiang Province, China, were conducted from September to October 2022 and from September to October 2023 by the Ningbo Center for Disease Control and Prevention (CDC). The overall educational level in Ningbo is relatively high, with abundant resources and balanced resource sharing. In general, approximately 940 and 1,227 students study at a junior high school and a senior high school, respectively. A multistage, stratified cluster sampling procedure was used to select the target samples, encompassing socio-economic status (SES), demographics, health, and psychological aspects. In stage 1, schools were randomly selected from 10 districts and counties. Two junior high schools, two senior high schools, and one academic and vocational high school were selected from each district, whilst two high schools and one academic and vocational high school were selected from each county. In stage 2, two classes per grade were randomly selected from each school. The questionnaires were administered on a class-by-class basis. At least 80 students were selected from each grade, and more than 240 students were selected from each school. The details of the study design have been previously reported (21).

**Figure 1:**
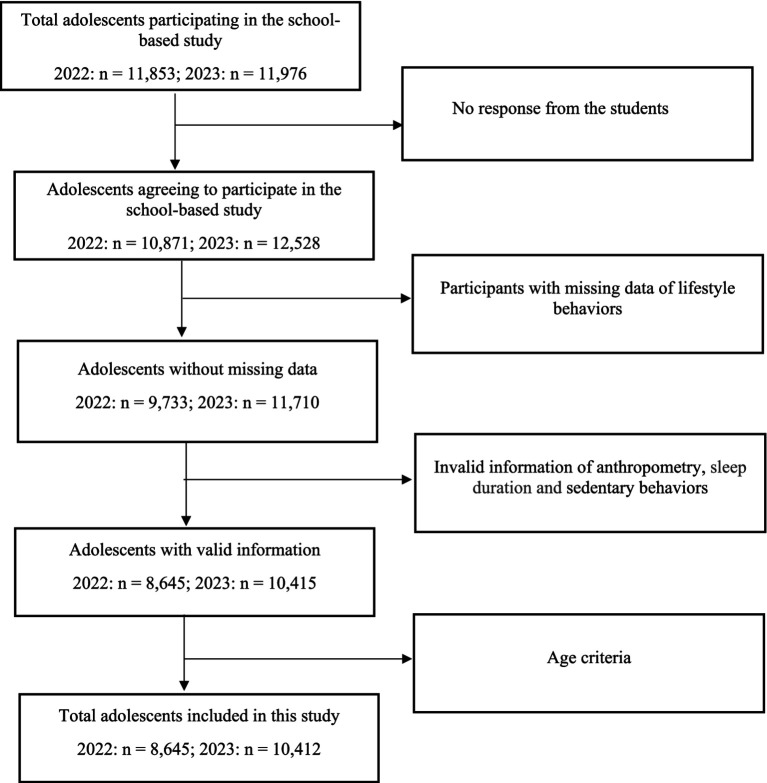
Flowchart of study population participating in this school-based study in 2022 and 2023.

A total of 23,829 high school students were contacted to participate in this study, of which 23,399 students participated in the repeated surveys, with a participation rate of 98.2% (Figure 1). Out of the 23,399 students, 1,956 students reported missing values for lifestyles, and 2,383 students’ information on anthropometry and lifestyles was invalid. Ultimately, 19,057 adolescents were included in this study.

The inclusion criteria were as follows: (1) students aged 11–19 years, (2) their ability to understand the questions in the questionnaire and complete the questionnaire independently, and (3) written informed consent obtained from a parent or legal guardian of the student for the student’s participation. The exclusion criteria were as follows: (1) students who participated in the survey without providing consent and (2) students who were ill (e.g., high fever) or had injuries (e.g., a broken leg) that affect the health examination, which was based on standard quality control of health examination for secondary school students (WS/T10020-2024), proposed by National Health Commission of the People’s Republic of China and Ministry of Education of the People’s Republic of China in 2021 (22).

Ethical approval for this study was obtained by Ningbo CDC (No. 202201) and the University of Nottingham Ningbo China, and followed the Declaration of Helsinki. Written informed consent was obtained from the parents or legal guardians of the students. All information related to students has been kept confidential.

### 2.2 Data collection

Before students filled in the questionnaires on health status and influencing factors for high school students in Ningbo, well-experienced researchers from Ningbo CDC provided an introduction and instructions for the survey and emphasised confidentiality. All students independently completed self-administered, anonymous questionnaires in their classrooms under the supervision of the researchers. All submitted information was double-checked for quality control by the researchers. Missing or misreported information was requested during the survey.

### 2.3 Measurement

#### 2.3.1 Assessment of depressive symptoms

The Chinese version of the Centre for Epidemiological Studies-Depression (CES-D) is a 20-item questionnaire that assesses the status of DSs for the past 7 days, which was previously validated and has been widely used on Chinese students with good reliability and validity (23–26). Students reported the frequency of each symptom that they have experienced during the past week. The CES-D has four response options with a score ranging from 0 to 3 for each item: 0—rarely or none of the time (<1 day), 1—some or little of the time (1–2 days), 2—moderately or much of all the time (3–4 days), and 3—most or almost all the time (5–7 days). The total score ranges from 0 to 60, with high scores indicating greater DSs (27) and a cutoff value of 16, identifying individuals with DSs (28).

#### 2.3.2 Assessment of lifestyle behaviours

The level of PA was assessed using the question: ‘How many days were you able to do at least 60 min of MVPA over the past week?’. PA was categorised into two groups: (1) < 3 days/week as a low level of MVPA and (2) ≥ 3 days/week as a high level of MVPA.

ST was assessed using the question: ‘How long did you use electronic devices over the past week, including watching television, using computers, and playing video games?’. The time spent on ST amongst Chinese adolescents was recorded as: (1) < 3 h/day as appropriate screen-based sedentary behaviour (SSB) and (2) ≥ 3 h/day as excessive ST (18).

All students reported their average SLD every night for the past week. SLD was categorised based on recommendations proposed by the National Sleep Foundation (29). Due to the few cases of very short and long SLD, SLD was categorised into two groups: (1) short SLD and (2) sufficient SLD.

#### 2.3.3 Covariates

During the survey, body weight and height were measured by experienced researchers and public health specialists. The BMI z-score was calculated to standardise the BMI values across sex and age groups (30). Body weight status was classified as underweight (UW), normal weight (NW), overweight (OW), and obesity (OB), using sex- and age-specific reference data from the WHO (31).

Students reported information on demographics [sex, age (≤12, 13–15, ≥16), school type (junior high school and senior high school/vocational high school), and residence area (urban, urban–rural junction, and rural areas)], family structure (single-parent, both-parent, grandparents, parents and grandparents, and others), whether they ever smoked (yes and no), and whether they ever consumed alcohol (yes and no).

### 2.4 Statistical analysis

In this study, we pooled two survey samples for the final analysis. Means with standard deviation (SD), and numbers with percentages are presented for continuous variables and categorical variables, respectively. Statistical differences in the percentages and mean values between non-DSs and DSs were tested using Pearson’s chi-square test (χ^2^) and Student’s *t*-test, respectively.

A sensitivity analysis was performed to validate the CES-D for Chinese adolescents. A Cronbach’s alpha above 0.85 indicates good internal reliability of the CES-D for Chinese adolescents (28). In this study, a Cronbach’s alpha of 0.91 confirmed that the CES-D questionnaire has high reliability among adolescents.

A binary logistic regression analysis was used to analyse the crude association between DSs and MVPA, ST, and SLD. A multivariate analysis was used to examine independent associations between MVPA, ST, and SLD and DSs after adjusting for demographics (sex, age groups, area of residence, family structure, and school type), smoking, alcohol consumption, health status or comorbidities (heart diseases, anaemia, hepatitis, disability, hypertension, diabetes, and allergic diseases), BMI, and the other two lifestyle behaviours.

The association between DSs and combined patterns of MVPA, ST, and SLD was investigated by the multivariate analysis via two models: (1) a crude model and (2) an adjusted model, adjusting for demographics, smoking, alcohol consumption, health status, or comorbidity and BMI. In these models, a healthy combined pattern of high MVPA, appropriate ST, and sufficient SLD was used as the reference group.

The results were considered statistically significant at a two-tailed level of 0.05. Statistical analysis was conducted using STATA statistical software package version 18 (2021).

## 3 Results

A total of 19,057 adolescents (52.1% boys) were included in the final analysis, of which 48.2 and 37.7% were aged 13–15 and ≥16 years, respectively; 46.8% were junior high school students; 69.9% lived in urban areas; and 59.8% were from both-parent families (Table 1). The percentages of demographics, lifestyle, body weight, and disease history (anaemia and allergic diseases) significantly differed between the non-DS and DS groups. Differences in the mean values of age, weight, and BMI were higher amongst adolescents with DSs than amongst those without DSs.

**Table 1:** General characteristics of adolescents.

	Total (n)	Non-depressive symptoms (*n* = 15,953) n (%)	Depressive symptoms (*n* = 3,104) n (%)	Prevalence of depression symptoms (%)	p*
Sex					
Male	9,937 (52.1)	8,499 (53.3)	1,438 (46.3)	14.5	<0.001
Female	9,120 (47.9)	7,454 (46.7)	1,666 (53.7)	18.3	
Age					
≤12 years	2,697 (14.2)	2,409 (15.1)	288 (9.3)	10.7	<0.001
13–15 years	9,184 (48.2)	7,754 (48.6)	1,430 (46.1)	15.6	
≥ 16 years	7,176 (37.7)	5,790 (36.3)	1,386 (44.7)	19.3	
School type					
Junior high	8,920 (46.8)	7,673 (48.1)	1,247 (40.2)	14.0	<0.001
Senior high or academic and vocational high school	10,137 (53.2)	8,280 (51.9)	1,857 (59.8)	18.3	
Family structure					
Single-parent	2,168 (11.4)	1,741 (10.9)	427 (13.8)	19.7	<0.001
Both-parent	11,405 (59.8)	9,626 (60.3)	1,779 (57.3)	15.6	
Grandparents	1,175 (6.2)	940 (5.9)	235 (7.6)	20.0	
Parents and grandparents	4,127 (21.7)	3,492 (21.9)	635 (20.5)	15.4	
Others	182 (0.96)	154 (0.97)	28 (0.90)	15.4	
Area of residence					
Urban	13,326 (69.9)	10,884 (68.2)	2,442 (78.7)	18.3	
Urban–rural junction/rural areas	5,731 (30.1)	5,069 (31.8)	662 (21.3)	11.6	<0.001
Ever smoked in lifetime (Yes)	462 (2.4)	270 (1.7)	192 (6.2)	41.6	<0.001
Ever drank alcohol in lifetime (Yes)	2,213 (11.6)	1,440 (9.0)	773 (24.9)	34.9	<0.001
Body weight status					
Underweight	2,926 (15.4)	2,459 (15.4)	467 (15.0)	16	0.128
Normal weight	11,144 (58.5)	9,273 (58.1)	1,871 (60.3)	16.8	
Overweight	3,333 (17.5)	2,815 (17.6)	518 (16.7)	15.5	
Obesity	1,654 (8.7)	1,406 (8.8)	248 (8.0)	15	
Disease history					
Heart disease	11 (0.06)	9 (0.06)	2 (0.06)	18.2	0.865
Anaemia	119 (0.62)	83 (0.52)	36 (1.2)	30.3	<0.001
Hypertension	10 (0.05)	8 (0.05)	2 (0.06)	20	0.751
Diabetes	2 (0.01)	1 (0.01)	1 (0.03)	50	0.197
Allergic diseases	81 (0.43)	60 (0.38)	21 (0.68)	25.9	0.019

Mean (SD)					
Age (years)	15.0 (1.8)	14.9 (1.8)	15.3 (1.7)		<0.001
Weight (kg)	58.2 (14.0)	58.1 (13.9)	58.7 (14.3)		0.032
Height (cm)	165.2 (9.0)	165.2 (9.0)	165.3 (8.7)		0.565
BMI	21.1 (4.0)	21.1 (4.0)	21.3 (4.1)		0.011

SD: standard deviation.

*Significant difference between non-depression and depression by categories and mean values was examined using Pearson’s chi-square test (χ^2^) and Student’s *t*-test, respectively.

DSs were prevalent in a total of 16.3% and were significantly more common in girls than in boys (Table 1). The prevalence of DSs increased significantly with age and was much higher amongst adolescents living in urban areas. Amongst adolescents, 41.6% of those who smoked and 34.9% of those who consumed alcohol had DSs.

Differences in the prevalence of DSs for the lifestyle factors of MVPA, ST, and SLD are shown in Table 2. The prevalence of DSs was significantly higher amongst adolescents with short SLD, excessive ST, and low MVPA.

**Table 2:** Prevalence of depressive symptoms stratified by moderate-to-vigorous physical activity, screen-based sedentary behaviour, and sleep duration.

	Total (n)	Non-depressive symptoms (*n* = 15,953) n (%)	Depressive symptoms (*n* = 3,104) n (%)	Prevalence of depressive symptoms (%)	p*
Moderate-to-vigorous physical activity					
Low	8,669	7,100 (44.5)	1,569 (50.5)	18.1%	<0.001
High	10,388	8,853 (55.5)	1,535 (49.5)	14.8%	
Screen-based sedentary behaviour					
Appropriate	17,241	14,632 (91.7)	2,609 (84.1)	15.1%	<0.001
Excessive	1,816	1,321 (8.3)	495 (15.9)	27.3%	
Sleep duration					
Short	12,150	9,902 (62.1)	2,248 (72.4)	18.5%	<0.001
Sufficient	6,907	6,051 (37.9)	856 (27.6)	12.4%	

*Significant difference between non-depression and depression was examined using Pearson’s chi-square test (χ^2^).

In the unadjusted model, low MVPA, excessive ST, and short SLD were independently associated with high odds of developing DSs (Table 3). After controlling for demographics, smoking, alcohol consumption, health status, and BMI, low MVPA, excessive ST, and short SLD were independently associated with greater odds of DSs in the adjusted model (all *p* < 0.001).

**Table 3:** Independent associations of moderate-to-vigorous physical activity, screen-based sedentary behaviour, and sleep duration with adolescents’ depressive symptoms.

Independent lifestyle behaviours	Unadjusted model^a^			Adjusted model^a^^,^^b^		
	OR	95% CI	*p*	OR	95% CI	*p*
Moderate-to-vigorous physical activity						
High	1			1		<0.001
Low	1.3	1.2,1.4	<0.001	1.3	1.2,1.4	
Screen-based sedentary behaviour						
Appropriate	1			1		
Excessive	2.1	1.9,2.4	<0.001	2.2	1.9,2.4	<0.001
Sleep duration						
Sufficient	1			1		<0.001
Short	1.6	1.5,1.8	<0.001	1.7	1.5,1.8	

OR, odds ratio; CI, confidence interval.

a Unadjusted model: crude model; adjusting demographics, smoking, alcohol consumption, health status, and BMI.

b Each independent lifestyle behaviour was adjusted for the other two lifestyle behaviours.

In the crude model, all combined patterns were associated with higher odds of DSs (Table 4). After controlling for demographics, smoking, alcohol consumption, health status or comorbidities, and BMI, all combined patterns remained significant in the adjusted model. The unhealthiest combined pattern of low level of MVPA, excessive ST, and short SLD was associated with high odds of DSs (OR:4.2, 95% CI:3.3, 5.2, *p* < 0.001), followed by the combination of high MVPA, excessive ST, and short SLD (OR:3.8, 95% CI:3.1, 4.8, *p* < 0.001), the combination of low MVPA, excessive ST, and sufficient SLD (OR:2.5, 95% CI:1.9, 3.2, *p* < 0.001), and the combination of high MVPA, excessive ST, and sufficient SLD (OR:2.4, 95% CI:1.8, 3.1, *p* < 0.001).

**Table 4:** Association between combined patterns of moderate-to-vigorous physical activity, screen-based sedentary time, sleep duration, and adolescents’ depressive symptoms.

Combined patterns	MVPA	ST	Sleep duration	Unadjusted model^a^			Adjusted modela		
				OR	95% CI	*p*	OR	95% CI	*p*
Group 1	High	Appropriate	Sufficient	1			1		
Group 2	High	Appropriate	Short	1.7	1.5,1.9	<0.001	1.7	1.4,1.9	<0.001
Group 3	High	Excessive	Sufficient	2.5	1.9,3.2	<0.001	2.4	1.8,3.1	<0.001
Group 4	High	Excessive	Short	3.8	3.0,4.7	<0.001	3.8	3.1,4.8	<0.001
Group 5	Low	Appropriate	Sufficient	1.3	1.1,1.5	0.003	1.3	1.1,1.5	0.006
Group 6	Low	Appropriate	Short	2.1	1.9,2.4	<0.001	2.2	1.9,2.4	<0.001
Group 7	Low	Excessive	Sufficient	2.6	2.0,3.4	<0.001	2.5	1.9,3.2	<0.001
Group 8	Low	Excessive	Short	4.2	3.4,5.2	<0.001	4.2	3.3,5.2	<0.001

MVPA, moderate-to-vigorous physical activity; ST, screen-based sedentary time; OR, odds ratio; CI, confidence interval.

a Unadjusted model: crude model; adjusted model: adjusting for demographics, smoking and alcohol consumption, health status, and BMI.

## 4 Discussion

This is the first representative study to investigate the association between combined patterns of MVPA, ST, and SLD and DSs amongst Chinese adolescents aged 11–19 years. Our findings indicate that MVPA, ST, and SLD are independently associated with DSs, and that unhealthy combinations of these lifestyle factors increase the likelihood of DSs.

The overall prevalence of DSs in our study was slightly higher than that reported in our previous study in 2022 (15.3%), reflecting the current increase in the incidence of reported DSs in Ningbo, Zhejiang Province, China (10). The prevalence of DSs was lower than the estimated prevalence for adolescents in Zhejiang Province (22.4%) and at the national level in China (19.9%) (6, 32) and was far lower than that for adolescents from Hubei Province (34.0%) (33). This significant discrepancy in the prevalence of DSs may be attributable to regional factors, as Ningbo is amongst the most affluent cities in China (6). Compared to other countries, the prevalence of DSs in our study was lower than that reported amongst adolescents in the USA (20.1%) and South Korea (26.0%) (34, 35). Consistent with previous studies, the prevalence of DSs in our study was higher among girls than boys (20, 32, 34), suggesting that girls are more likely to experience DSs during adolescence. In addition, the prevalence of DSs increased with age, which is in line with previous findings (10, 32). This trend may be explained by greater academic pressure and the impact of social media on social adjustment in later adolescence.

Adolescents’ lifestyle behaviours are important modifiable health risk factors for the development of DSs, as this period is critical for cognitive and psychosocial growth (9). With rapid economic development, adolescents’ lifestyles have transitioned towards lower levels of PA, shorter SLD, and an increased use of electronic devices (12, 36, 37). In line with previous studies, our findings also revealed individual associations between DSs and PA, ST, and SLD (10, 15, 16).

Our results, consistent with those reported in previous studies, showed significant associations between combined patterns of lifestyle behaviours and DSs (38, 39). These findings indicate that the co-existence of several risk behaviours is associated with an increased risk of DSs (39). It has been suggested that the risk of developing DSs is approximately two times higher among individuals who have combined patterns of unhealthy lifestyle behaviours than amongst those who do not (40). A combination of low MVPA, excessive ST, and short SLD had the strongest effect on DSs amongst Chinese adolescents in our study. Given the limited available evidence on associations between different combined patterns of lifestyle behaviours and DSs, only one systematic review has indicated favourable associations between meeting all recommendations and better mental health indicators amongst children and adolescents from 12 countries (17), which supports our findings.

In our study, combined patterns of high or low MVPA, short or sufficient SLD, and excessive ST were associated with higher odds of DSs than a healthy combined pattern of these lifestyle behaviours. In our study, excessive ST had a greater effect on increasing DSs than either MVPA or SLD. The highest prevalence of DSs was observed in the subgroup of excessive ST (27.3%). This finding is consistent with previous studies focusing on mental illness among adolescents, which reported that adolescents with excessive ST were nearly twice as likely to suffer from DSs than those with appropriate ST (18, 37). Existing evidence on the association between ST and DSs in adolescents remains limited due to restrictive age ranges and fewer reports from longitudinal studies. One previous study reported that excessive ST was longitudinally associated with a higher risk of DSs at 1-year follow-up (41). Possible mechanisms include the arousal of the central nervous system, sleep disturbances, poor metabolic health, and reduced PA, all of which may contribute to DSs and other mental health disorders (42, 43).

The association between the combined pattern of high/low levels of MVPA, short/sufficient SLD, along with excessive SSB and DSs, suggested that the interaction between MVPA and SLD had a worse impact on the development of DSs. A school-based longitudinal survey found that the interaction between PA and SLD was a significant predictor of anxiety and DSs amongst Japanese adolescents (44). In addition, the combination of either sufficient SLD or adequate PA was reported to maintain good mental health amongst Japanese adolescents (44). A higher level of PA has been suggested to have beneficial effects on SLD, with a decrease in the occurrence of DSs (45). In our study, a low MVPA was associated with a greater likelihood of DSs than high MVPA was in adolescents. Similarly, engagement in no days of PA and a lack of participation in any PA were associated with greater odds of DSs than meeting the daily PA guidelines and engaging in daily PA behaviour (37). The levels of PA, considered an intermediary factor, were associated with increased cerebral blood flow, regulation of serum cortisol levels and metabolism, better neurotransmitter regulation, promotion of neuronal health, and optimisation of neurotrophins such as brain-derived neurotrophic factors (46), which may be related to the risk of DSs (47). In addition, PA may enhance social contact by improving the self-perception subdomains of sport competence, perceived strength, physical condition, physical attractiveness, and self-confidence (48).

Our results indicated that SLD may play a critical role in triggering the development of DSs and mental health problems amongst adolescents. In our study, short SLD was associated with an increased risk of DSs in adolescents, which is consistent with previous studies (21, 49). A cross-sectional study conducted on Japanese adolescents reported a reverse J-shaped relationship between SLD and the level of DSs (50). The possible causal mechanism for this association between SLD and DSs may be that short SLD in adolescents negatively affects neurotransmitter levels, which regulate mood and thoughts (51). In addition, a short SLD impairs cognitive function, resulting in psychological problems and mental wellbeing (52). Moreover, a short SLD could be associated with the activation of the hypothalamic–pituitary–adrenal and the sympathoadrenal–medullary axes, thus increasing the risk of mental health disorders (53).

This study used data sampled from a representative school-based study with a large sample size to investigate the association of the combined patterns of PA, ST, and SLD with DSs amongst Chinese adolescents. Although significant findings can provide evidence to fill a gap in the literature, this study still has several limitations. First, causality could not be inferred due to the observational study design. Second, self-reported information relied on potential recall and psychological biases, which may misreport or underestimate CES-D scores and PA levels, as well as the duration of ST and sleep. Therefore, it may affect the accuracy of the DS level and its association with combinations of MVPA, ST, and SLD. Furthermore, data on parents’ education and marriage were not collected. The lack of these factors was not adjusted for in the models; thus, in the end, it might have indirectly influenced the accuracy of the associations. Future well-designed studies are needed to explore longitudinal associations between the combined patterns of lifestyle behaviours and DSs in Chinese adolescents.

## 5 Conclusion

This study found that 16.3% of adolescents had DSs. MVPA, ST, and SLD were independently and in combination associated with DSs. Compared with the healthy combined pattern of high MVPA, appropriate ST, and sufficient SLD, the unhealthiest combination pattern of low MVPA, excessive ST, and short SLD was associated with the highest odds of DSs, followed by the combination of high MVPA, excessive ST, and short SLD; the combination of low MVPA, excessive ST, and sufficient SLD; and the combination of high MVPA, excessive ST, and sufficient SLD. Our results can be used as evidence to assist public health specialists in improving strategies for developing school-based health education programmes aimed at promoting lifestyles to prevent DSs and other mental disorders amongst Chinese adolescents. Future longitudinal studies are required to clarify the causal relationships between the combined patterns of these lifestyles and DSs amongst Chinese adolescents.

## Data Availability

The data is not publicly available due to privacy or ethical restrictions. If there is a reasonable request, it can be obtained from the corresponding authors.
